# A question of time

**DOI:** 10.7554/eLife.36786

**Published:** 2018-04-16

**Authors:** Peter Rodgers

**Affiliations:** 1 eLifeCambridgeUnited Kingdom

**Keywords:** Scientist and Parent, women in science, careers in science, grad school, postdoc

## Abstract

How does a scientist balance establishing a career and starting a family?

*Parents in the Pipeline*, a report published last year by the Center for WorkLife Law in San Francisco, starts with some good news: "Gone are the days where a tenured male professor leads research while his wife stays at home and raises the children." What follows, however, is not such good news: "Gone, too, are the days when postdocs worked non-stop for two years before quickly moving into a professorship of their own. The average postdoc today can’t postpone solving the puzzle of work-life fit until tenure [...]. Most postdocs are nearing 40 by the time they reach their first permanent position. To add to the challenge, parents of this generation feel the need to be more present for their children. For postdocs, the buzzers on their biological and research clocks are undeniable – and in conflict."

So, how does a scientist resolve the conflict between their biological and research clocks? The answer, according to the 20+ scientists interviewed by eLife for this collection of articles, is that there is no right or wrong time for a scientist to think about becoming a parent. Some of our interviewees were well established in their careers when they had their first child, but more were less established.

## Big decisions

"There is a saying, 'there is never a perfect time to have children', and I think this saying applies to graduate students as well," says Anne Hakim, a second-year graduate student at the University of Michigan and the mother of a three-year-old girl. "Despite the hurdles, I would not want to go through grad school any other way. Having kids makes me more focused and time-efficient, knowing I have to make every second away from my kid worth it." Hakim's husband is a fourth-year graduate student at Michigan.

Bede Portz is a postdoc at the University of Pennsylvania who became the father of twins while he was a graduate student at Pennsylvania State University. What advice does he have for other scientists who are thinking of having children? "Do it – the magnitude of the joy it has brought me far outweighs any career challenges", he says. "I look back on career-related reasons we waited to have kids and they all seem so trivial in hindsight. Having children during graduate school will be difficult, it may even add time to your grad school career, but it can be done!"

"I got married at the beginning of my PhD and we always intended to have a family, although not straight away," says Viki Male, who is a Sir Henry Dale Fellow at University College London. "My PhD was in reproductive immunology, so I saw a lot of women who struggled to conceive and for this reason I wanted to have at least my first child before turning 30." At the same time, Male didn’t want to start a family until she was an established scientist, with her own funding: "But as I approached the end of my first postdoc, at 27, I took a look at the career landscape and decided that I would never be running my own group before 30, so we decided to just go ahead."

Returning to science was more difficult than she expected: Male applied for lots of postdocs but didn’t get any of them, and almost left science as a result. "However," she recalls, "the desperation of that time did push me to apply for things I assumed I didn’t have enough experience for. I wrote the application for my current fellowship sitting on a park bench, in between pushing the buggy and, to my surprise, it was funded!"

So, what would Male – who now has her own lab and two daughters – say to other scientists who are wondering when to start a family? "There is never a good time to have children. If you do it in the middle of a project, you lose productivity. If you do it at the end of a project, you risk a period of unemployment. So you might as well do it at the time that feels right for you personally. For me, the rewards of family life were greater – and the impact on my productivity less – than I expected, so I’m glad I took the plunge."

"For me, the rewards of family life were greater – and the impact on my productivity less – than I expected, so I’m glad I took the plunge."

Lotte Meteyard and her partner were never sure if they wanted to have children. However, as she approached 35, having become an associate professor in the neuropsychology of language at the University of Reading, they decided to try and their child is now approaching three years old. "There is never going to be a perfect time," she says. "If you want to have children, then go ahead and try. Make sure you have open and frank discussions with your partner about how childcare duties will be arranged and what each of you needs. You will have to compromise, and you should keep having this discussion as things change."

## Sound advice

Our interviewees also had lots of advice and tips for would-be and new parents. "Reduce future commitments in advance," says Adrian Liston of the VIB research institute in Leuven. "A baby is not a surprise, you have months of notice. You are going to have a major restriction on your time, so start saying 'no' in advance." Saying no more, says Liston, will help you to save your time for "the stuff that actually matters to your career – mainly, big grants and major papers."

Alexis Barr, who is looking for an independent group leader position after eight years as a postdoc at the Institute of Cancer Research in London, stresses the importance of establishing collaborations with other researchers. "Collaborations make a huge difference to my working life", says Barr, who has two young sons. "Having various people working towards different aspects of a project means that the project is constantly being driven forward, even if your part is slower at times – for example, when you are on parental leave."

If time is something that always seems to be in short supply for new parents, the same is not true about advice. Anne-Laura van Harmelen, a Royal Society Dorothy Hodgkin Fellow at Cambridge University, and Rogier Kievit have two children: "The one thing that makes parenthood different from many other aspects of life is that people seem disinhibited from giving unsolicited advice about everything," says Kievit, who is a programme leader in the MRC Cognition and Brain Sciences Unit in Cambridge. "So, if I might ironically violate my own advice by giving advice: Try your best to not listen too much."

## Note

This Feature Article is part of the Scientist and Parent collection.

